# Association of Bariatric Surgery With Rates of Kidney Function Decline Using Multiple Filtration Markers

**DOI:** 10.1001/jamanetworkopen.2020.14670

**Published:** 2020-09-04

**Authors:** Alex R. Chang, G. Craig Wood, Xin Chu, Aditya Surapaneni, Morgan E. Grams

**Affiliations:** 1 Kidney Health Research Institute, Geisinger, Danville, Pennsylvania; 2 Department of Population Health Sciences, Geisinger, Danville, Pennsylvania; 3 Obesity Institute, Geisinger, Danville, Pennsylvania; 4 Welch Center for Prevention, Epidemiology, and Clinical Research, Johns Hopkins University, Baltimore, Maryland; 5 Divison of Nephrology, Johns Hopkins University, Baltimore, Maryland

## Abstract

This cohort study examines the association of bariatric surgery with rates of kidney function decline.

## Introduction

Kidney function is typically assessed by estimated glomerular filtration rate using creatinine (eGFR_cr_), a marker heavily influenced by muscle mass.^1^ Patients who undergo bariatric surgery have large changes in muscle mass, which may confound changes in eGFR_cr_ over time. Other kidney filtration markers, such as cystatin C, β-2 microglobulin (β_2_m), and β-trace protein (βTP), are less affected by muscle mass but are rarely used clinically.^2^ The objective of this study was to examine whether bariatric surgery was associated with a slower rate of kidney function decline, evaluating change in eGFR calculated using creatinine, cystatin C, β_2_m, βTP, and combination of creatinine and cystatin C, with the latter believed to be the most accurate GFR estimating equation in the general population and in individuals who have undergone bariatric surgery.^1,3^

## Methods

This cohort study was approved by Geisinger Health System’s institutional review board. The informed consent requirement was waived per Geisinger policy, as participants had previously consented to a biobanking research study.^4^ This study followed the Strengthening the Reporting of Observational Studies in Epidemiology (STROBE) reporting guideline for cohort studies.

This observational, matched cohort study included adults aged 18 years or older at Geisinger Health System with body mass index (BMI; calculated as weight in kilograms divided by height in meters squared) 35 or higher with 2 biobanked serum samples, stored at −80 °C. Baseline evaluations were conducted from January 1, 2005, to December 31, 2009, and follow-up examinations were conducted from January 1, 2015, to December 31, 2017.

Patients who underwent bariatric surgery were matched 1 to 1 with participants who never underwent bariatric surgery based on sex, self-reported race, preoperative BMI (±10), age (±5 years), and eGFR_cr_ (±10%). Each blood serum sample was measured for creatinine (enzymatic assay; Roche, isotope-dilution mass spectroscopy-traceable calibration; precision, 2.8%-2.9%), cystatin C (turbidometric assay; Gentian; precision, 3.2%-4.3%), β_2_m (latex agglutination; Roche; precision, 3.2%-5.1%), and βTP (immunonephelometric; Siemens; precision, 7.4%-10.6%) at the University of Minnesota. We used Chronic Kidney Disease Epidemiology equations to estimate GFR.^3,5^

The primary outcome was rate of decline in combined creatinine-cystatin C eGFR (eGFR_cr-cyc_). Secondary outcomes included rate of decline in eGFR_cr_, eGFR_cyc_, eGFR_β2m_, and eGFR_βTP_. Mixed-effects models with random intercepts and slopes were used to compare eGFR trajectories between groups. We also examined whether the association differed by baseline eGFR_cr-cyc_, and conducted subgroup analyses by baseline eGFR_cr-cyc_ levels. All analyses were completed using Stata/MP statistical software version 15.1 (StataCorp) from September 14, 2019, to June 7, 2020. We considered 2-sided *P* < .05 as statistically significant.

## Results

Of 311 patients who underwent bariatric surgery and met inclusion criteria, a total of 144 patients (47.9 [10.2] years; 126 [87.5%] women) were matched with 144 participants (mean [SD] age, 48.5 [10.7] years; 126 [87.5%] women) who did not undergo bariatric surgery. Participants who underwent bariatric surgery vs those who did not were well-matched with no significant differences in mean (SD) eGFR_cr-cyc_ (82.6 [19.9] mL/min/1.73 m^2^ vs 82.6 [18.2] mL/min/1.73 m^2^), hypertension (74 participants [51.4%] vs 71 participants [49.3%]), or diabetes (59 participants [41.0%] vs 59 participants [41.0%]) (Table). Baseline mean (SD) BMI was higher in the surgery group (46.2 [6.4] vs 44.1 [6.1]; *P* = .01). Mean eGFR_cr-cyc_ decline rates were –0.41 (95% CI, –0.74 to –0.08) mL/min/1.73 m^2^ per year over a mean (SD) follow-up of 9.2 (1.4) years for the surgery group, and –1.44 (95% CI, –1.76 to –1.11) mL/min/1.73 m^2^ per year over a mean (SD) follow-up of 8.2 (1.1) years for the no surgery group (Table). Thus, bariatric surgery was associated with a 1.02 (95% CI, 0.56 to 1.49) mL/min/1.73 m^2^ per year slower decline in eGFR_cr-cyc_. Results were qualitatively similar using eGFR_cr_, eGFR_cyc_, eGFR_β2m_, and eGFR_βTP_ or when adjusted for baseline BMI (Table). Bariatric surgery was associated with a greater slowing of eGFR decline among those with lower baseline eGFR (*P* for continuous interaction = .04) (Figure).

**Table.** Characteristics and Differences in Rate of eGFR Decline Between Individuals Who Underwent Bariatric Surgery and Those Who Did Not

Characteristics	Individuals, No. (%)		Difference (95% CI)	*P* value
	Surgery (n = 144)	No surgery (n = 144)		
Age, mean (SD), y	47.9 (10.2)	48.5 (10.7)	NC	.62
Women	126 (87.5)	126 (87.5)	NC	>.99
Black race	1 (0.7)	1 (0.7)	NC	>.99
Weight, mean (SD), kg	126.2 (22.1)	118.6 (19.3)	NC	.002
BMI, mean (SD)	46.2 (6.4)	44.1 (6.1)	NC	.01
Hypertension	74 (51.4)	71 (49.3)	NC	.72
Diabetes	59 (41.0)	59 (41.0)	NC	>.99
eGFR_cr-cyc_, mean (SD), mL/min/1.73 m^2^	82.6 (19.9)	82.6 (18.2)	NC	>.99
Time from baseline to follow-up serum samples, median (IQR), y	9.17 (8.12 to 9.96)	8.20 (7.46 to 8.80)	NC	<.001
Weight change from baseline to follow-up, median (IQR), kg	–27.5 (–37.9 to –19.9)	–1.7 (–9.1 to 7.6)	NC	<.001
Rate of eGFR decline, mL/min/1.73 m^2^/y, mean (95% CI)^a^				
eGFR_cr-cyc_	–0.41 (–0.74 to –0.08)	–1.44 (–1.76 to –1.11)	1.02 (0.56 to 1.49)	<.001
eGFR_cr_	–0.86 (–1.16 to –0.56)	–1.49 (–1.84 to –1.15)	0.63 (0.17 to 1.08)	.01
eGFR_cyc_	–0.06 (–0.41 to 0.29)	–1.40 (–1.73 to –1.07)	1.34 (0.85 to 1.82)	<.001
eGFR_β2m_	–0.18 (–0.43 to 0.06)	–0.79 (–1.02 to –0.57)	0.60 (0.26 to 0.94)	.001
eGFR_βTP_	–0.18 (–0.41 to 0.05)	–0.97 (–1.19 to –0.76)	0.79 (0.47 to 1.10)	<.001

Abbreviations: BMI, body mass index (calculated as weight in kilograms divided by height in meters squared); βTP, β-trace protein; β_2_m, β-2 microglobulin; cr, creatinine; cyc, cystatin C; eGFR, estimated glomerular filtration rate; IQR, interquartile range; NC, not calculated.

^a^ Calculated using Chronic Kidney Disease Epidemiology equations.

**Figure.**
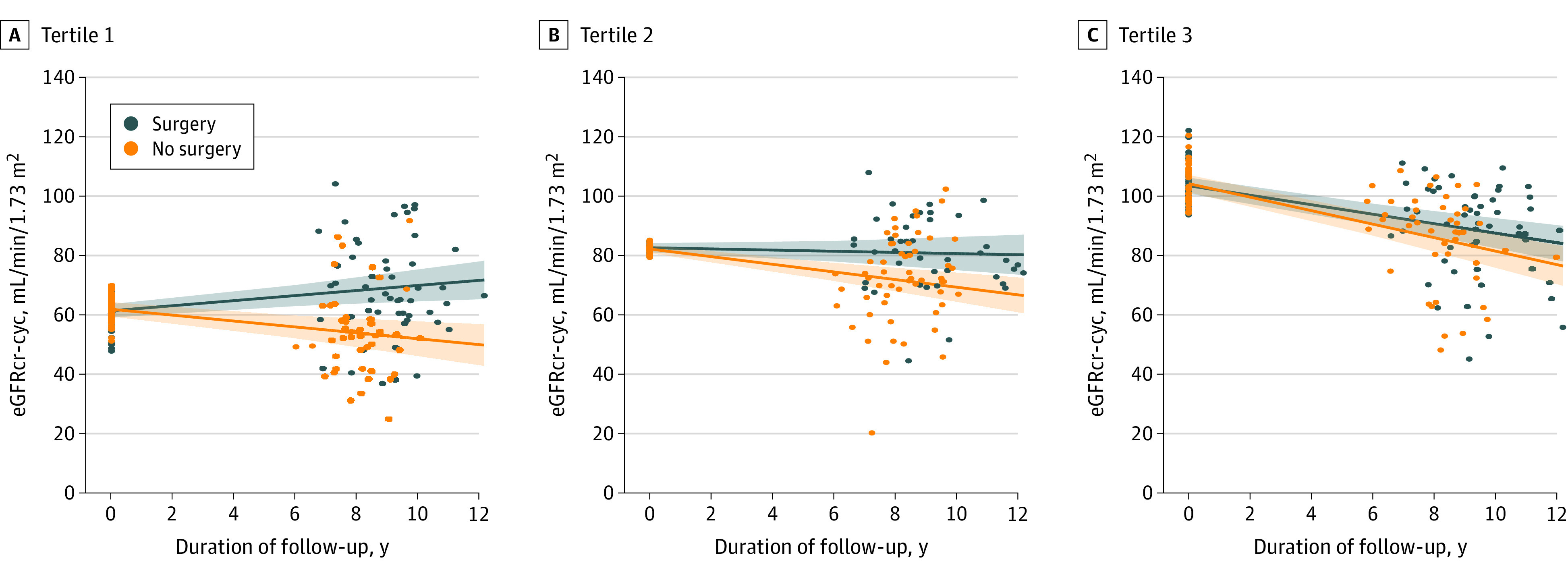
Rates of Kidney Function Decline Stratified by Baseline Kidney Function

## Discussion

To our knowledge, this cohort study is the first study to confirm a beneficial association of bariatric surgery with long-term kidney function trajectory using multiple filtration markers. The marked association between bariatric surgery and improved kidney function trajectory in patients at lower baseline kidney function was consistent with other studies that only examined eGFR_cr_.^6^ Limitations of our study include a requirement to survive the interval period, potential selection bias, and other residual confounding. Since patients who undergo bariatric surgery must demonstrate sustained weight loss prior to the procedure, they may have had better adherence to healthy lifestyle behaviors and medication therapy. Strengths of this study include the use of multiple filtration factors to show consistency of the association and the long-term time interval. In conclusion, bariatric surgery was associated with long-term improvement in kidney function trajectory, assessed using multiple filtration markers.
